# Seasonality in Respiratory Syncytial Virus Hospitalizations and Immunoprophylaxis

**DOI:** 10.1001/jamahealthforum.2023.1582

**Published:** 2023-06-30

**Authors:** Jennifer D. Kusma, Michelle L. Macy, Larry K. Kociolek, Matthew M. Davis, Sriram Ramgopal

**Affiliations:** 1 Division of Advanced General Pediatrics and Primary Care, Department of Pediatrics, Ann & Robert H. Lurie Children’s Hospital of Chicago, Chicago, Illinois; 2 Mary Ann & J. Milburn Smith Child Health Outcomes, Research, and Evaluation Center, Stanley Manne Children’s Research Institute, Ann & Robert H. Lurie Children’s Hospital of Chicago, Chicago, Illinois; 3 Division of Infectious Disease, Department of Pediatrics, Ann & Robert H. Lurie Children’s Hospital of Chicago, Chicago, Illinois; 4 Department of Pediatrics, Northwestern University Feinberg School of Medicine, Chicago, Illinois; 5 Division of Emergency Medicine, Department of Pediatrics, Ann & Robert H. Lurie Children’s Hospital of Chicago, Chicago, Illinois

## Abstract

This cross-sectional study assesses whether current guidance on respiratory syncytial virus supports the current epidemiologic characteristics, treatment, and hospitalization patterns in respiratory syncytial virus.

## Introduction

Respiratory syncytial virus (RSV) frequently causes hospitalization,^1^ particularly among children with hemodynamically significant congenital heart disease, chronic lung disease, or prematurity (<29 weeks’ gestation).^2^ Immunoprophylaxis administration to high-risk children during active RSV circulation periods is cost-effective.^3^ American Academy of Pediatrics (AAP) guidelines recommended that high-risk children receive RSV immunoprophylaxis with monoclonal antibodies.^2^ Insurers typically cover RSV immunoprophylaxis annually (November-March) in accordance with RSV seasonality.^2,4^ Since the COVID-19 pandemic, RSV has been characterized by unexpected interseasonal spikes. During the 2021 summer RSV surge, the AAP recommended RSV immunoprophylaxis for high-risk children,^5^ but adoption has varied among states and insurers. Interseasonal spikes and policy lag create periods in which RSV immunoprophylaxis provides no protection, which potentially undermines its cost-benefit ratio. We investigated patterns in RSV-related hospitalizations and RSV-immunoprophylaxis administration timing to account for changing RSV epidemiological characteristics to inform policy discussions between clinicians and public and commercial insurance.

## Methods

This retrospective cross-sectional study identified children younger than 2 years who were hospitalized for RSV between July 2017 and November 2022 using the Pediatric Health Information System (PHIS). The Ann & Robert H. Lurie Children’s Hospital of Chicago Institutional Review Board deemed this study exempt from review and waived the informed consent requirement because it used deidentified data. We followed the STROBE reporting guideline.

*International Statistical Classification of Diseases, Tenth Revision, Clinical Modification* diagnosis codes (eMethods in Supplement 1) were used to identify RSV-related hospitalizations and children with RSV-immunoprophylaxis eligibility. We defined *RSV seasons* as months during which RSV-related hospitalizations exceeded by 3 SDs mean monthly hospitalization counts (June-September; >350 per month).^6^ Using χ^2^ test, we compared hospitalizations between 2017 to 2020 and 2021 to 2022 RSV seasons and in interseasons.

Two-sided *P* < .05 indicated statistical significance. Analysis was performed with R 4.1.2 (R Core Team).

## Results

We identified 4 RSV seasons with 109 185 RSV-related hospitalizations (Figure) among 104 898 children (median [IQR] age, 4.7 [2.0-10.6] months; 45 931 girls [43.8%], 58 947 boys [56.2%]). Seasonal spikes between 2017 and 2019 began in October. High-risk children represented a larger proportion of hospitalizations during interseasons (Table). Children with public insurance constituted the largest proportion of hospitalizations, and even larger during interseasons.

**Figure.**
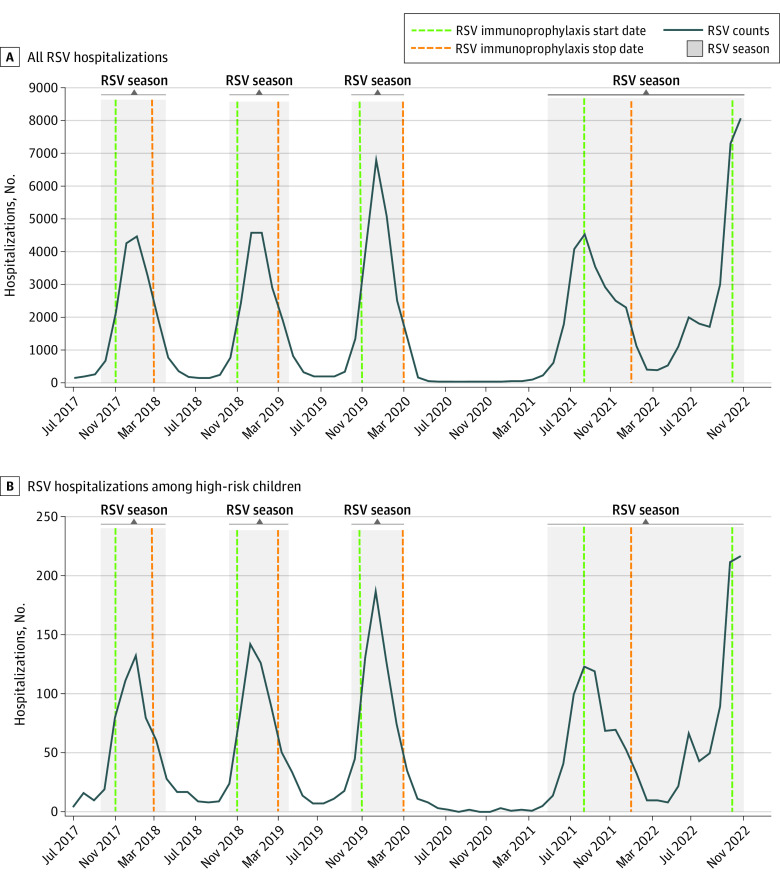
Respiratory Syncytial Virus (RSV) Hospitalization Patterns With RSV Seasons and RSV Immunoprophylaxis From 2017 to 2022 High risk was defined as having hemodynamically significant congenital heart disease, chronic lung disease of prematurity, or less than 29 weeks’ gestation. In the 2021 to 2022 season, states had variable start and stop dates for RSV immunoprophylaxis.

**Table.** Comparison of RSV Hospitalizations by Season From 2017 to 2022

Variable	Participants, No. (%)			*P* value
	Combined 2017- 2019 seasons (n = 56 579)	2021-2022 season (n = 49 475)	Interseason (n = 3131)	
Age categories, mo				
<2	15 775 (27.9)	11 621 (23.5)	740 (23.6)	<.001
2 to <4	11 332 (20.0)	9531 (19.3)	599 (19.1)	
4 to <6	7200 (12.7)	6124 (12.4)	373 (11.9)	
6 to <9	6660 (11.8)	6271 (12.7)	420 (13.4)	
9 to <12	4619 (8.2)	4466 (9.0)	315 (10.1)	
12 to <24	10 993 (19.4)	11 462 (23.2)	684 (21.8)	
Sex^a^				
Female	24 807 (43.8)	21 507 (43.5)	1336 (42.7)	.69
Male	31 763 (56.1)	27 958 (56.5)	1794 (57.3)	
Race and ethnicity^b^				
Hispanic	12 966 (22.9)	10 768 (21.8)	908 (29.0)	<.001
Non-Hispanic Black	8954 (15.8)	8454 (17.1)	740 (23.6)	
Non-Hispanic White	27 332 (48.3)	24 054 (48.6)	1116 (35.6)	
>1 or other^c^	5082 (9.0)	4189 (8.5)	255 (8.1)	
None documented	2245 (4.0)	2010 (4.1)	112 (3.6)	
Census region				
Midwest	16 297 (28.8)	14 302 (28.9)	640 (20.4)	<.001
Northeast	4800 (8.5)	5629 (11.4)	178 (5.7)	
South	24 235 (42.8)	23 318 (47.1)	1992 (63.6)	
West	11 247 (19.9)	6226 (12.6)	321 (10.3)	
RSV immunoprophylaxis– eligible^d^	1668 (2.9)	1355 (2.7)	185 (5.9)	<.001
CHD	322 (0.6)	222 (0.4)	38 (1.2)	<.001
Chronic lung disease of prematurity	1128 (2.0)	900 (1.8)	135 (4.3)	<.001
Prematurity: <29 weeks’ gestation	565 (1.0)	576 (1.2)	56 (1.8)	<.001
Insurance type				
Public	34 940 (61.8)	28 440 (57.5)	2143 (68.4)	<.001
Private	19 783 (35.0)	18 801 (38.0)	883 (28.2)	
Other^e^	1542 (2.7)	1821 (3.7)	86 (2.7)	
Unknown	314 (0.6)	413 (0.8)	19 (0.6)	
ICU admission	17 800 (31.5)	13 115 (26.5)	1217 (38.9)	<.001
Mechanical ventilation	6794 (12.0)	4179 (8.4)	495 (15.8)	<.001

Abbreviations: CHD, congenital heart disease; ICU, intensive care unit; RSV, respiratory syncytial virus.

^a^ Sex data were unavailable for 20 patients.

^b^ Race and ethnicity data were obtained from local hospital records, which may include self-reported or electronic health record–based data.

^c^ Other race and ethnicity included Alaska Native, American Indian, Asian, Pacific Islander, and other.

^d^ Defined as having hemodynamically significant congenital heart disease, chronic lung disease of prematurity, or less than 29 weeks’ gestation.

^e^ Other insurance type included charity, no bill, self-pay, and other payer.

Seasonal hospitalization pattern from 2017 to 2019 (starting in November, ending in March) aligned with RSV immunoprophylaxis availability. However, the 2021 RSV season started in May (579 hospitalizations per month). Public and commercial insurance in most states did not approve RSV immunoprophylaxis until September or later.^5^ Although RSV immunoprophylaxis ended between January and March 2022 and restarted in October or November 2022, RSV season was continuous, with monthly hospitalizations larger than the interseasonal threshold. The 2021 to 2022 RSV season consisted of 45% of all hospitalizations over the study period.

## Discussion

We found that RSV-related hospitalizations deviated from expected seasonality pattern during the pandemic. The AAP guidelines encouraged out-of-season RSV immunoprophylaxis authorization.^5^ The RSV surge was missed by the time insurance covered RSV immunoprophylaxis. Many high-risk children could not receive RSV immunoprophylaxis during peak months.

Study limitations included reliance on hospital administrative data to identify RSV cases. Generally, few hospitals submit ambulatory data to PHIS, and children are inconsistently tested for RSV in the outpatient setting.

This analysis suggests that current RSV-immunoprophylaxis guidance based on historical seasonality does not align with current RSV-related hospitalization patterns. To maximize benefits of RSV immunoprophylaxis, preventing hospitalization and death among high-risk children,^2^ policies must be responsive to atypical epidemiological patterns. Surveillance is not a universal public health activity. Even among jurisdictions that perform RSV surveillance, data may not be pediatric-specific and/or available in real time to inform policy decisions. Existing public health–hospital relationships and data infrastructure could be leveraged for real-time pediatric surveillance to guide RSV immunoprophylaxis initiation and cessation. Additionally, policymakers may establish flexible models that are responsive to timely regional RSV epidemiological data, maximizing coverage during periods of high RSV activity and minimizing unnecessary doses when RSV activity is low.

## Notes

Supplement 1.
eMethods.

Supplement 2.
Data Sharing Statement
