# Supplementary material for: Seasonality in Respiratory Syncytial Virus Hospitalizations and Immunoprophylaxis
Source: JAMA Health Forum. 2023 Jun 30;4(6):e231582. doi: 10.1001/jamahealthforum.2023.1582 (PMC10314303; doi:10.1001/jamahealthforum.2023.1582)
Supplement: Supplement 1. — eMethods. [file jamahealthforum-e231582-s001.pdf]

Supplementary Online Content

Kusma JD, Macy ML, Kociolek LK, Davis MM, Ramgopal S. Seasonality in respiratory syncytial virus hospitalizations and immunoprophylaxis. *JAMA Health Forum.* 2023;4(6):e231582. doi:10.1001/jamahealthforum.2023.1582

eMethods

This supplementary material has been provided by the authors to give readers additional information about their work.

© 2023 Kusma JD et al. *JAMA Health Forum*.

eMethods

The Pediatric Health Information System (PHIS) is a dataset that includes hospital administrative data from geographically diverse US children's hospitals that are members of the Children's Hospital Association (Overland Park, KS). Data quality and reliability are assured through a joint effort between the Children's Hospital Association and participating hospitals. For the present analysis, we included data from 40 children's hospitals with complete data for all study years. RSV-related hospitalizations were defined using International Classification of Disease, 10th revision, Clinical Modification (ICD-10-CM) diagnoses codes of RSV Pneumonia (J12.1), RSV Bronchitis (J20.5), and RSV Bronchiolitis (J21.0).[1] ICD-10-CM codes were also used to identify RSV-IP eligible children with hemodynamically significant congenital heart disease, chronic lung disease of prematurity, or prematurity.[2]

1. Cai W, Tolksdorf K, Hirve S, et al. Evaluation of using ICD-10 code data for respiratory syncytial virus surveillance. *Influenza Other Respir Viruses*. Nov 2020;14(6):630-637. doi:10.1111/irv.12665
2. American Academy of Pediatrics (AAP). Updated guidance for palivizumab prophylaxis among infants and young children at increased risk of hospitalization for respiratory syncytial virus infection. *Pediatrics*. Aug 2014;134(2):415-20. doi:10.1542/peds.2014-1665

© 2023 Kusma JD et al. *JAMA Health Forum*.
