# Supplementary material for: Seasonality in Respiratory Syncytial Virus Hospitalizations and Immunoprophylaxis
Source: JAMA Health Forum. 2023 Jun 30;4(6):e231582. doi: 10.1001/jamahealthforum.2023.1582 (PMC10314303; doi:10.1001/jamahealthforum.2023.1582)
Supplement: Supplement 2. — Data Sharing Statement [file jamahealthforum-e231582-s002.pdf]

Data Sharing Statement

Kusma. Seasonality in Respiratory Syncytial Virus Hospitalizations and Immunoprophylaxis. *JAMA Health Forum*. Published June 30, 2023. doi:10.1001/jamahealthforum.2023.1582

Data
Data available: No
